# How to Induce and Recognize Facial Expression of Emotions by Using Past Emotional Memories: A Multimodal Neuroscientific Algorithm

**DOI:** 10.3389/fpsyg.2021.619590

**Published:** 2021-05-10

**Authors:** Michela Balconi, Giulia Fronda

**Affiliations:** ^1^International Research Center for Cognitive Applied Neuroscience (IrcCAN), Catholic University of the Sacred Heart, Milan, Italy; ^2^Research Unit in Affective and Social Neuroscience, Department of Psychology, Catholic University of the Sacred Heart, Milan, Italy

**Keywords:** emotion recognition, facial expressions recognition, emotional memories, human-computer interaction, multimodal, neuroscientific techniques

## Introduction: Facial Expression Production and Recognition as a Ict Challenge

Emotion expression production and recognition play a decisive and central role in individuals’ life. The consideration and the investigation of emotions result to be especially important allowing to comprehend individuals’ emotional experiences and empathic mechanisms, representing driving knowledge for brain-computer interfaces (BCI), through the implementation of emotional patterns into artificial intelligence tools and computers, and for in-deep comprehension of psychopathology (Balconi et al., 2015a).

This article aims to allow the investigation of the neurophysiological correlates and characteristics associated with individuals’ facial expressions production and recognition, considering emotional responses provoked by internal cues based on autobiographic memories, called “self-induced by memories.”

Indeed, as reported by Adolphs (2002), the human brain represents most effectively emotional data through the connection of information between different cerebral areas that allow to state and recognize emotional expressions from different stimuli, as visual or auditory ones. The human brain represents emotional data connecting facial, voice, and movement expressions with individuals’ past experiences. Moreover, the use of different neuroscientific techniques, as positron emission tomography (PET), functional magnetic resonance imaging (fMRI), and magnetoencephalography (MEG), allow observing the involvement of specific cerebral regions in different emotional expressions, providing a map of the emotional brain activation (Balconi and Lucchiari, 2007; Balconi and Pozzoli, 2007; Deak, 2011; Kassam et al., 2013). Specifically, neuroimaging measures are used as input to Affective Computing technologies (Frantzidis et al., 2010).

Different studies postulate the existence of discrete emotions, such as happiness, fear, anger, sadness, from which the other emotional states would derive (Ekman, 1999). The theory of discrete emotions has been criticized by the Circumplex Model of Affect (Russell, 1980), that describe and label emotions on the base of two dimensions: valence and arousal. Multimodal information are integrated by the human brain generating an integrated representation of different auditory and visual stimuli (Balconi and Carrera, 2011; Barros and Wermter, 2016).

An important role is also played by facial motion in emotion perception and recognition. By providing unique information about the direction, quality, and speed of motion, dynamic stimuli enhance coherence in the identification of affect, lead to stronger emotion judgments, and facilitate the differentiation between posed and spontaneous expressions (Krumhuber et al., 2017; Oh et al., 2018; Goh et al., 2020). In this regard, also new technologies have introduced important innovations in face detection and recognition (Canedo and Neves, 2019). For example, sensors that may provide extra information and help the facial recognition systems to detect emotion in both static images and video sequences (Samadiani et al., 2019).

However, despite the vast theoretical differences emerged in previous studies, it is commonly shared that emotional states and consequent responses to external stimuli are influences by arousal and valence (Balconi and Carrera, 2011; Balconi and Molteni, 2016), conceptualized in different ways as tension and energy, positive or negative affect, approach and withdrawal, and valence and arousal (Russell, 1980; Eysenck, 1990; Lang et al., 1997; Watson et al., 1999). In particular, valence refers to the pleasantness or unpleasantness of individuals’ emotional states; while, arousal refers to individuals’ perception of activation or not. Considering these two dimensions, therefore, each emotional state experienced by individuals can be defined according to a two-dimensional model, including respectively, the valence and arousal axis.

The emotions of individuals, therefore, represent overlapping experiences that are cognitively interpreted in order to identify the responses and neurophysiological changes in the valence and arousal dimensions organized based on different eliciting factors, as different contexts and stimuli, autobiographical memories, and semantic representation or behavioral responses (Russell, 2003; Balconi and Vanutelli, 2016; Balconi et al., 2017).

In this perspective, we may represent emotions as communication signals, as they allow individuals to implement sensorimotor responses congruent with external stimuli by attributing meaning to internal and external information. This process addresses both the individual’s body and the external environment, allowing the attribution of emotional meaning to the states experienced. Therefore, from a functional perspective, emotions are used to recognize and categorize some individual states in different social contexts.

Actually, facial expression recognition is well considered in the fields of computer vision, pattern recognition, artificial intelligence, and has drawn extensive attentions owing to its potential applications to natural human-computer interaction (HCI), human emotion analysis, interactive video, image indexing and retrieval.

Indeed, the emotional states’ recognition from face patterns and expression allows us to comprehend and satisfy the user’s needs facilitating human-machine interaction, especially when only emotional states are used to communicate with others (Kanchanadevi et al., 2019; Volynets et al., 2020). This shows how the fundamental role of emotions in individuals’ cognition (LeDoux, 1998) symbolizes a defying topic in Information Communication Technology (ICT) useful to respond to the high request, implementing machines able to assist individuals with several psychological and physical disorders or difficulties at a cognitive, social, or communicative level (Esposito and Jain, 2016).

Also, integration between emotional memories (EM) and emotional expression by faces is an interesting topic. In particular, the role of EM in emotional facial production and recognition has been observed by some studies that have focused mainly on patterns of emotional recognition in specific contexts. Indeed, EM allow the use of internal emotional models developed through the individuals’ past life experiences to decode others’ emotions expressed through mimic facial patterns. This mechanism is permitted simulating the emotional states expressed in oneself (Dimberg et al., 2000; Heberlein and Atkinson, 2009; Niedenthal et al., 2010; Wearne et al., 2019).

## The Technology-Based Recognition of Facial Patterns

In the last years, the interest regarding the investigation of emotion through electroencephalography (EEG) is increased, thanks to the possibility provided by this tool to label and recognize facial expressions. Furthermore, compared to neuroimaging techniques, as fMRI, MEG, and PET, the EEG is configured as a low cost and easy to use technique thanks to the development of current wireless EEG systems. Recent studies have observed the advantages of using EEG to investigate emotions, providing the measurement of cerebral changes in high- and low-frequency band activity and early and late latencies with an excellent temporal resolution and offering a full overview of the emotional processes. Indeed, brain activity changes depict in a sequential way the dynamicity of individuals’ emotional responses variations, that are not fully accessible using neuroimaging techniques (Balconi and Canavesio, 2014; Balconi et al., 2014, 2015b).

However, considering the quick temporal evolution of emotional responses and the interconnection of different cerebral areas and neural networks involved in emotional processing, neuroimaging techniques, that provide a good temporal and spatial resolution, could be useful for the investigation of emotional facial expression and recognition. In particular, the fNIRS, consisting of a non-invasive and easy-to-use technique, provides a sufficient temporal resolution to investigate event-related hemodynamic changes (Elwell et al., 1993). Indeed, in the last years, fNIRS has been used to investigate emotional responses in various contexts (Koseki et al., 2013; Balconi and Molteni, 2016). Furthermore, the portability, the lack of restrictions, and fNIRS replicability allow, compared to other neuroimaging techniques, to impose lower physical and psychological burdens on participants.

Moreover, the combined use of fNIRS and EEG allows obtaining information about the neural and hemodynamic correlates of brain activity. In addition to electrophysiological and neuroimaging techniques, autonomic ones provide an integration of the central measures, contributing to the integration of the previous order of measures. Finally, EMG allows measuring the zygomaticus major and the corrugator supercilii muscle activity, which characterize the facial autonomic response to emotional stimuli, representing predictive markers of emotional behavior (Fridlund and Cacioppo, 1986).

## Ways to Elicit and Recognize Facial Expression

As reported by different studies, several techniques are used for the elicitation of emotional responses and expressions. Among these, primary methods of emotional elicitation consist of movies and pictures with highly emotional content. In particular, as demonstrated by Westermann et al. (1996), watching movies result to be the best procedure to elicit positive or negative emotions. Therefore, researchers have proposed different databases containing affective video-clips (Balconi et al., 2009; Chambel et al., 2011) or pictures and sounds with high emotional content to cause emotional responses and expression (Balconi and Pozzoli, 2005). Among these, two of the most used databases of audio and visual emotional elicitation stimuli are the International Affective Digitized Sounds (IADS) (Bradley and Lang, 2007) and the International Affective Picture System (IAPS) (Lang et al., 2008).

However, emotions’ elicitation and recognition can also be produced by recalling in mind past experiences. Indeed, also integration between EM and emotion expression by faces is an interesting topic. In particular, for the elicitation of self-generated emotions, individuals were asked to re-experience personal life episodes, positive or negative connoted, and marked by different emotions (Damasio et al., 2000; Kassam et al., 2013). The elicitation of self-generated emotions through memories could be a way to drive BCI independently.

Recently, databases included stimuli media belonging to different modalities of emotional elicitation and expression have been suggested (Gunes and Piccardi, 2006; Grimm et al., 2008; Fanelli et al., 2010; Koelstra et al., 2012; Soleymani et al., 2012; Abadi et al., 2015; Katsigiannis and Ramzan, 2018) and implemented through the use of the recognition of patterns of signals derived from different modalities. The databases previously described have the limit of not being generalizable regarding the modalities of measurement and elicitation of emotions, since they have considered certain strategies of signal classification and their results. Due to this structure, these databases result to be very useful to conduct comparisons between different elaboration strategies or classification on the same data, but they make impossible the conduction of transversal studies and the comparison between data of the same subjects collected from different imaging or activation modalities. This allows us to observe how the existing and used databases have limitations to a complete and generalizable investigation of emotional elicitation responses and mechanisms. In fact, in the first place, these databases use partial methods for emotional investigation. Furthermore, different reference models and methods used in previous research are not directly comparable. Besides, these methods do not allow to distinguish the different cognitive components that are involved and that are fundamental in emotional processing, such as memory and its contribution.

Despite the existence of different emotional elicitation techniques, the creation of algorithmic related to emotions’ induction and recognition appears to be difficult because individuals’ emotional elicitation includes different components, as behavioral, psychological, and cognitive ones. This leads to the consideration of data regarding individuals with different features collected by different methods and techniques, thus including a large number of evidence belonging to different subjects in structured databases.

## New Perspective to Induce and Recognize Facial Expression of Emotions

In light of what is reported in the previous paragraph, the use of existing methods and databases for the induction and the recognition of emotion should be integrated with new databases that consider the collection of different parameters using self-induced stimuli. For example, the steps for creating a database for the induction and recognition of emotion based on self-induced stimuli will be presented below, consisting of recalling past autobiographical events of EM. Specifically, the first step requires collecting autobiographical experiences of individuals through mnemonic recall using semi-structured interviews of autobiographical events with a positive, negative and neutral valence. We clearly explained the subjects the scope, the experimental phases and content and the detailed procedure of the present experiment. An explicit consent to participate (and to withdraw from the experiment in any time) was required for each participant.

The second step requires creating specific algorithms for the formulation of linguistic codes (short utterances memory induction) for the encoding of the participants’ autobiographical memories. The utterances were vocally reproduced and then submitted to a specific vocal analysis. Indeed specific parameters (such as F0, speech profile, intensity, temporal parameter – i.e., locutory duration and pause etc.) were checked before the experimental phase. It was made to avoid any vocal effect and to make more neutral the linguistic stimuli.

Finally, the third step requires the evocation of emotional experiences through previously created emotional cues, coded in a personalized linguistic way, after a specific time interval. The time interval we adopted was considered based on previous studies on the memory effect related to long-lasting effect. Indeed we intend to work with long-term memories and avoid potential transient effect due only to working memory. For this reason, this time interval was adopted. The advantage of using a database of this type allows the use of an automated and recognized procedure for the induction and the recognition of emotion and it allows explicit reference to fundamental processes, such as memory, in emotional recognition. Furthermore, this database could also be used in the clinical setting, especially in the case of deficits or syndromes related to emotional memory, in order to investigate behavioral and neurophysiological responses with eliciting stimuli, through the support of different neuroscience tools.

## Discussion: A New Procedure to Induce and Recognize Facial Expression: The Role of Autobiographical Memories

Therefore, given emotions’ importance and their various pathways, it is necessary to design a structured multimodal database, based on memories of past experiences able to induce facial expression modulated by specific valence/arousal. As anticipated in the previous paragraph, we suggest some pathways to create specific procedure and dataset to induce emotional response and to active a better recognition of facial expression.

In the first place, the collection of EM must be produced and chosen from a positive, negative, and neutral valence typology. For the creation of the EM database, three specific steps are requested. In particular, the first step includes the free recall based on positive, negative, and neutral past autobiographical events collected through semi-structured interview administered to participants by an expert researcher. Specifically, the recalling of autobiographical memories occurred freely, but participants were asked to provide certain information regarding each event, describing the specific moment, the duration and the place of this event in their life.

Moreover, the second step includes the codification of participants’ autobiographical memories by expert judges, that reports them in sentences using a specific algorithm to encode participants’ memories into linguistic code (brief utterances - - bu – memory inducing) able to elicit past autobiographical events positive, negative and neutral connoted. The homogeneity of these linguistic codes has been verified at a grammatical and structural level. In this way, 25 sentences were created for valence (positive, negative, and neutral). Finally, the third step requires the guided recall by listening to emotional cues, previously created, after a specific time interval, consisting of 5 days, from the first step. These guided recalls are based on the initial memory and are coded in a personalized way and transposed in a communicable and objective way using a linguistic code (see Table 1).

**TABLE 1:** Procedural steps for the creation of the EM database.

**Step**	**Procedure**	**Characteristics**	**Example**
First step	Free recall based on positive, negative, and neutral past autobiographical events	This step consists of the free recall of autobiographical events based on positive, negative, and neutral individuals’ past events. These autobiographical events of individuals are collected through semi-structured interviews conducted by experienced researchers. Specifically, this step required participants to freely recall specific past life events by recalling certain information such as the duration of the event throughout the day, the location of the event and the specific time of day when it took place	
Second step	Codification of participants’ autobiographical memories	This step consists of the collection of autobiographical memories previously produced by each individual. These memories are subsequently coded by expert judges who transpose them into linguistic codes through specific algorithms (short statements induction of the memory recorded by the experimenter). The grammatical and structural homogeneity of these linguistic codes has been verified (linguistic code consisting of subject + verb + direct object) to create 25 positive, 25 negative, and 25 neutral sentences ad hoc for each individual	
Third step	Guided recall by listening to emotional cues	This step consists of the guided recall based on listening to statements, reproduced in auditory format, after 5 days compared to the first step. In particular, the statements have been divided into three categories concerning valence and arousal. These dimensions were assessed using a 9-point Likert scale.	Positive high arousal utterances: “*Lo spettacolo riceve davvero molti complimenti*,” “*The show really receives a lot of compliments*,” “*L’esame ottiene un ottimo risultato*,” “*The exam gets an excellent result*”; Positive low arousal utterances: “*La nonna cucina i biscotti preferiti*,” “*Grandmather cooks the favorite cookies*,” “*Il papà racconta le storie d’infanzia*,” “*Dad tells childhood stories*”; Negative high arousal utterances “*L’esame non superato genera grande sconforto*,” “*The failed exam generates discouragement*,” “*Il colloquio di lavoro ha esito davvero negativo*,” “*The job interview greatly fails*”; Negative low arousal utterances “*La sessione di laurea viene rimandata*,” “*The graduation session is postponed*,” “*Gli altri compagni passano l’esame*,” “*The other companions pass the exam*”; Neutral: “*Un’amica arriva la mattina sotto casa*,” “*An acquaintance arrives in the house in the morning*,” “*Il bus arriva alla fermata*,” “*The bus arrives at the stop*.”

The significant effect induced by subjective memories is the crucial point of this dataset: it was created to be able to induce specific emotional responses directly evoked by the subjective recall. Since we verified each cue’s exact significance (personal memory and then utterance), we are sure that this dataset can induce specific psychological answers in a subject. This procedure may bypass the limitation of the previous dataset, in which “impersonal” cues are used to elicit an emotional experience or an emotional recognition process. In this last case, we are not sure that the “impersonal” dataset is really able to provoke exactly that emotion. Only by using personal cues, related to the subjective experience (their memories), we can be sure that the real emotional meaning is intrinsic to the cues. This emotional meaning has a high psychological power in terms of emotion induction, since EM were previously demonstrated to be the most significant event able to induce emotions in a subject.

The neurophysiological correlates of facial expression associated with individuals’ emotional response elicited by EM could be collected with the simultaneous use of EEG, fNIRS, and autonomic measures. When we associate the EM to facial expressions this link is potentially of high impact to facilitate the facial recognition.

These measures as suggested to better support a multimodal acquisition in order to have a picture of central and peripheral components.

In addition this procedure allows possible future clinical applications in case of subjects with specific consciousness impairment (such as DOC or locked-in syndrome patients) where the use of memories could be a valid alternative to the impossibility to communicate their emotional states by language: this is a possible future development for BCI (brain-computer interface) based on EM and thoughts which are supposed to be able to activate specific physiological activation in the absence of an explicit communication (when impaired), and, for this reason, this tool could become a valid way to improve the quality of life of specific categories of patients.

## Author Contributions

MB wrote the first draft and each section of the manuscript. MB and GF contributed to manuscript final writing and revision, read, and approved the submitted version.

## Conflict of Interest

The authors declare that the research was conducted in the absence of any commercial or financial relationships that could be construed as a potential conflict of interest.

## References

[B1] Abadi M. K. Subramanian R. Kia S. M. Avesani P. Patras I. Sebe N. (2015). DECAF: MEG-based multimodal database for decoding affective physiological responses. *IEEE Trans. Affect. Comput.* 6 209–222. 10.1109/TAFFC.2015.2392932

[B2] Adolphs R. (2002). Neural systems for recognizing emotion. *Curr. Opin. Neurol.* 12 169–177. 10.1016/S0959-4388(02)00301-X 12015233

[B3] Balconi M. Brambilla E. Falbo L. (2009). Appetitive vs. defensive responses to emotional cues. autonomic measures and brain oscillation modulation. *Brain Res.* 1296 72–84. 10.1016/j.brainres.2009.08.056 19703433

[B4] Balconi M. Canavesio Y. (2014). High-frequency rTMS on DLPFC increases prosocial attitude in case of decision to support people. *Soc. Neurosci.* 9 82–93. 10.1080/17470919.2013.861361 24279315

[B5] Balconi M. Carrera A. (2011). Cross-modal integration of emotional face and voice in congruous and incongruous pairs: the P2 ERP effect. *J. Cogn. Psychol.* 23 132–139. 10.1080/20445911.2011.473560

[B6] Balconi M. Finocchiaro R. Canavesio Y. (2014). Reward-system effect (BAS rating), left hemispheric “unbalance”(alpha band oscillations) and decisional impairments in drug addiction. *Addict. Behav.* 39 1026–1032. 10.1016/j.addbeh.2014.02.007 24629323

[B7] Balconi M. Grippa E. Vanutelli M. E. (2015a). What hemodynamic (fNIRS), electrophysiological (EEG) and autonomic integrated measures can tell us about emotional processing. *Brain Cogn.* 95 67–76. 10.1016/j.bandc.2015.02.001 25721430

[B8] Balconi M. Lucchiari C. (2007). Consciousness and emotional facial expression recognition: Subliminal/supraliminal stimulation effect on N200 and P300 ERPs. *J. Psychophysiol*. 21 100–108. 10.1027/0269-8803.21.2.100

[B9] Balconi M. Molteni E. (2016). Past and future of near-infrared spectroscopy in studies of emotion and social neuroscience. *J. Cogn. Psychol.* 28 129–146. 10.1080/20445911.2015.1102919

[B10] Balconi M. Pozzoli U. (2005). Morphed facial expressions elicited a N400 ERP effect: A domain-specific semantic module? *Scand. J. Psychol.* 46 467–474. 10.1111/j.1467-9450.2005.00478.x 16277647

[B11] Balconi M. Pozzoli U. (2007). Event-related oscillations (EROs) and event-related potentials (ERPs) comparison in facial expression recognition. *J. Neuropsychol*. 1 283–294. 10.1348/174866407X184789 19331021

[B12] Balconi M. Tirelli S. Frezza A. (2015b). Event-related potentials (ERPs) and hemodynamic (functional near-infrared spectroscopy, fNIRS) as measures of schizophrenia deficits in emotional behavior. *Front. Psychol.* 6:1686. 10.3389/fpsyg.2015.01686 26579058 PMC4630975

[B13] Balconi M. Vanutelli M. E. (2016). Vocal and visual stimulation, congruence and lateralization affect brain oscillations in interspecies emotional positive and negative interactions. *Soc. Neurosci.* 11 297–310. 10.1080/17470919.2015.1081400 26256040

[B14] Balconi M. Vanutelli M. E. Grippa E. (2017). Resting state and personality component (BIS/BAS) predict the brain activity (EEG and fNIRS measure) in response to emotional cues. *Brain Behav.* 7:e00686. 10.1002/brb3.686 28523228 PMC5434191

[B15] Barros P. Wermter S. (2016). Developing crossmodal expression recognition based on a deep neural model. *Adapt. Behav* 24 373–396. 10.1177/1059712316664017 27853349 PMC5098700

[B16] Bradley M. M. Lang P. J. (2007). “The international affective picture system (IAPS) in the study of emotion and attention,” in *Handbook of Emotion Elicitation and Assessment* 29, eds James A. C. John J. B. A. (Oxford: Oxford university press), 10.1037/0021-9010.69.1.85

[B17] Canedo D. Neves A. J. (2019). Facial expression recognition using computer vision: a systematic review. *Appl. Sci.* 9:4678. 10.3390/app9214678

[B18] Chambel T. Oliveira E. Martins P. (2011). “Being happy, healthy and whole watching movies that affect our emotions,” in *Proceeding of the International Conference on Affective Computing and Intelligent Interaction*, (Berlin: Springer), 35–45. 10.1007/978-3-642-24600-5_7

[B19] Damasio A. R. Grabowski T. J. Bechara A. Damasio H. Ponto L. L. B. Parvizi J. (2000). Subcortical and cortical brain activity during the feeling of self-generated emotions. *Nat. Neurosci.* 3:1049. 10.1038/79871 11017179

[B20] Deak A. (2011). Brain and emotion: cognitive neuroscience of emotions. *Rev. Psychol.* 18 71–80.

[B21] Dimberg U. Thunberg M. Elmehed K. (2000). Unconscious facial reactions to emotional facial expressions. *Psychol. Sci.* 11 86–89. 10.1111/1467-9280.00221 11228851

[B22] Ekman P. (1999). “Basic emotions,” in *Handbook of Cognition and Emotion*, eds Dalgleish T. Power M. (New York, NY: John wiley & sons Ltd), 10.1017/S0140525X0800349X

[B23] Elwell C. E. Owen-Reece H. Cope M. Wyatt J. S. Edwards A. D. Delpy D. T. (1993). “Measurement of adult cerebral haemodynamics using near infrared spectroscopy,” in *Monitoring of Cerebral Blood Flow and Metabolism in Intensive Care*, eds Unterberg A. W. Schneider G. H. Lanksch W. R. (Vienna: Springer), 74–80. 10.1007/978-3-7091-9302-0_13 8310866

[B24] Esposito A. Jain L. C. (2016). “Modeling emotions in robotic socially believable behaving systems,” in *Toward Robotic Socially Believable Behaving Systems-Volume I*, eds Esposito A. Jain L. (Cham: Springer), 9–14. 10.1007/978-3-319-31056-5_2

[B25] Eysenck H. J. (1990). The biopsychology of mood and arousal. *Pers. Indiv. Differ.* 11:993. 10.1016/0191-8869(90)90284-X

[B26] Fanelli G. Gall J. Romsdorfer H. Weise T. Van Gool L. (2010). A 3-D audio-visual corpus of affective communication. *IEEE Trans. Multimed.* 12 591–598. 10.1109/TMM.2010.2052239

[B27] Frantzidis C. A. Bratsas C. Papadelis C. L. Konstantinidis E. Pappas C. Bamidis P. D. (2010). Toward emotion aware computing: an integrated approach using multichannel neurophysiological recordings and affective visual stimuli. *IEEE T. Inf. Technol. in Biomed.* 14 589–597. 10.1109/TITB.2010.2041553 20172835

[B28] Fridlund A. J. Cacioppo J. T. (1986). Guidelines for human electromyographic research. *Psychophysio* 23 567–589. 10.1111/j.1469-8986.1986.tb00676.x 3809364

[B29] Goh K. M. Ng C. H. Lim L. L. Sheikh U. U. (2020). Micro-expression recognition: an updated review of current trends, challenges and solutions. *Vis. Comput.* 36 445–468. 10.1007/s00371-018-1607-6

[B30] Grimm M. Kroschel K. Narayanan S. (2008). “The vera am mittag german audio-visual emotional speech database,” in *Proceeding of the IEEE International Conference on*, (IEEE), 865–868. 10.1109/ICME.2008.4607572

[B31] Gunes H. Piccardi M. (2006). “A bimodal face and body gesture database for automatic analysis of human nonverbal affective behavior,” in *Proceeding of the Pattern Recognition, 2006. ICPR 2006. 18th International Conference on*, Vol. 1 (IEEE), 1148–1153.

[B32] Heberlein A. S. Atkinson A. P. (2009). Neuroscientific evidence for simulation and shared substrates in emotion recognition: beyond faces. *Emotion. Rev*. 1 162–177. 10.1177/1754073908100441

[B33] Kanchanadevi P. Subhashree K. Kalpana C. (2019). Recognition of facial expression by utilizing feed forward artificial neural networks. *J. Crit. Rev*. 7:2020. 10.31838/jcr.07.04.164

[B34] Kassam K. S. Markey A. R. Cherkassky V. L. Loewenstein G. Just M. A. (2013). Identifying emotions on the basis of neural activation. *PLoS One* 8:e66032. 10.1371/journal.pone.0066032 23840392 PMC3686858

[B35] Katsigiannis S. Ramzan N. (2018). DREAMER: a database for emotion recognition through EEG and ECG Signals from wireless low-cost off-the-shelf devices. *IEEE J. Biomed. Health Inform.* 22 98–107. 10.1109/JBHI.2017.2688239 28368836

[B36] Koelstra S. Mühl C. Soleymani M. Lee J. S. Yazdani A. Ebrahimi T. (2012). DEAP: a database for emotion analysis; using physiological signals. *IEEE Trans. Affect. Comput.* 3 18–31. 10.1109/T-AFFC.2011.15

[B37] Koseki S. Noda T. Yokoyama S. Kunisato Y. Ito D. Suyama H. (2013). The relationship between positive and negative automatic thought and activity in the prefrontal and temporal cortices: a multi-channel near-infrared spectroscopy (NIRS) study. *J. Affect. Disord.* 151 352–359. 10.1016/j.jad.2013.05.067 23829998

[B38] Krumhuber E. G. Skora L. Küster D. Fou L. (2017). A review of dynamic datasets for facial expression research. *Emot. Rev.* 9 280–292. 10.1177/1754073916670022

[B39] Lang P. J. Bradley M. M. Cuthbert B. N. (1997). “International affective picture system (IAPS): technical manual and affective ratings,” in *NIMH Center for the Study of Emotion and Attention*, eds Lang P. J. Bradley M. M. Cuthbert B. N. 39–58. 10.1027/0269-8803/a000147

[B40] Lang P. J. Bradley M. M. Cuthbert B. N. (2008). *International Affective Picture System (IAPS): Affective Ratings of Pictures and Instruction Manual. Technical Report A-8.* 10.1016/j.epsr.2006.03.016

[B41] LeDoux J. (1998). *The Emotional Brain: The Mysterious Underpinnings of Emotional Life.* New York, NY: Simon and Schuster.

[B42] Niedenthal P. M. Mermillod M. Maringer M. Hess U. (2010). The simulation of smiles (SIMS) model: embodied simulation and the meaning of facial expression. *Behav. Brain Sci.* 33 417–433. 10.1017/S0140525X10000865 21211115

[B43] Oh Y. H. See J. Le Ngo A. C. Phan R. C. W. Baskaran V. M. (2018). A survey of automatic facial micro-expression analysis: databases, methods, and challenges. *Front. Psychol.* 9:1128. 10.3389/fpsyg.2018.01128 30042706 PMC6049018

[B44] Russell J. A. (1980). A circumplex model of affect. *J. Person. Soc. Psychol.* 39:1161. 10.1037/h0077714

[B45] Russell J. A. (2003). Core affect and the psychological construction of emotion. *Psychol. Rev.* 110:145. 10.1037/0033-295X.110.1.145 12529060

[B46] Samadiani N. Huang G. Cai B. Luo W. Chi C. H. Xiang Y. (2019). A review on automatic facial expression recognition systems assisted by multimodal sensor data. *Sensors* 19:1863. 10.3390/s19081863 31003522 PMC6514576

[B47] Soleymani M. Lichtenauer J. Pun T. Pantic M. (2012). A multimodal database for affect recognition and implicit tagging. *IEEE Trans. Aff. Comput.* 3 42–55. 10.1109/T-AFFC.2011.25

[B48] Volynets S. Smirnov D. Saarimäki H. Nummenmaa L. (2020). Statistical pattern recognition reveals shared neural signatures for displaying and recognizing specific facial expressions. *Soc. Cogn. Affect. Neurosci.* 15 803–813. 10.1093/scan/nsaa110 33007782 PMC7543934

[B49] Watson D. Wiese D. Vaidya J. Tellegen A. (1999). The two general activation systems of affect: structural evolutionary considerations, and psychobiological evidence. *J. Pers. Soc. Psychol.* 76:820. 10.1037/0022-3514.76.5.820

[B50] Wearne T. Osborne-Crowley K. Rosenberg H. Dethier M. McDonald S. (2019). Emotion recognition depends on subjective emotional experience and not on facial expressivity: evidence from traumatic brain injury. *Brain Inj.* 33 12–22. 10.1080/02699052.2018.1531300 30296178

[B51] Westermann R. Spies K. Stahl G. Hesse F. W. (1996). Relative effectiveness and validity of mood induction procedures: a meta-analysis. *Eur. J. Soc. Psychol* 26 557–580. 10.1002/(sici)1099-0992(199607)26:4<557::aid-ejsp769>3.0.co;2-4

